# HLA expression and HLA type associations in relation to EBV status in Hispanic Hodgkin lymphoma patients

**DOI:** 10.1371/journal.pone.0174457

**Published:** 2017-03-23

**Authors:** Luke B. Fletcher, Rianne N. Veenstra, Eric Y. Loo, Amie E. Hwang, Imran N. Siddiqi, Lydia Visser, Bouke G. Hepkema, Ilja M. Nolte, Anke van den Berg, Wendy Cozen, Arjan Diepstra

**Affiliations:** 1 Department of Preventive Medicine and Norris Comprehensive Cancer Center, Keck School of Medicine of the University of Southern California, University of Southern California, Los Angeles, California, United States of America; 2 Department of Pathology and Medical Biology, University of Groningen, University Medical Center Groningen, Groningen, the Netherlands; 3 Department of Pathology and Norris Comprehensive Cancer Center, Keck School of Medicine of the University of Southern California, University of Southern California, Los Angeles, California, United States of America; 4 Department of Laboratory Medicine, University of Groningen, University Medical Center Groningen, Groningen, the Netherlands; 5 Department of Epidemiology, University of Groningen, University Medical Center Groningen, Groningen, the Netherlands; University of North Carolina at Chapel Hill, UNITED STATES

## Abstract

A proportion of classical Hodgkin lymphomas harbor the Epstein Barr virus (EBV). We previously demonstrated that associations between Human Leukocyte Antigen (HLA) alleles and susceptibility to EBV+ classical Hodgkin lymphoma differ between European and Chinese populations. Data on Hispanic populations is missing. Here we examined the association between HLA type, tumor cell HLA expression and other characteristics in Hispanic Hodgkin lymphoma patients. Hispanic Hodgkin lymphoma patients diagnosed at the Los Angeles County-University of Southern California Medical Center from 2000–2012 were included (n = 65). Formalin-fixed paraffin-embedded tumor tissue was analyzed for EBV by in situ hybridization and for HLA class I and class II expression by immunohistochemistry. HLA typing was performed by *HLA-A* specific quantitative PCR of genomic DNA from tissue. Thirty patients (46%) had EBV+ tumors. Expression of HLA class I (p = 0.0006) was significantly associated with EBV+ tumor status in Hispanic patients, similar to Europeans and Chinese. A positive association between HLA class II expression and EBV+ tumor status, as present in large studies in Europeans, was not found (p = 0.06). The prevalences of the specific European HLA-A*01 risk and European HLA-A*02 protective types were not significantly associated with EBV+ tumors among these Hispanic patients, however numbers were too low to draw firm conclusions. The HLA-A*02:07 allele, that is associated with EBV+ Hodgkin lymphoma in Chinese, was absent. In conclusion, the association between EBV positivity in tumor cells and HLA class I expression appears to be consistent across different populations. Larger studies in Hispanics are needed to evaluate HLA allele susceptibility associations.

## Introduction

Classical Hodgkin lymphoma (cHL) is a malignant lymphoma of B cell origin defined by a pathognomonic giant malignant cell, the Hodgkin Reed-Sternberg (HRS) cell. It comprises 11% of malignant lymphomas and is the most common lymphoma of young adults. cHL is a unique malignancy as the majority of the tumor is composed of non-malignant cells surrounding the HRS cells [1].

Epidemiologically, cHL is composed of multiple disease subtypes with different risk patterns based on age [2], histology [3], and Epstein Barr Virus (EBV) status [4–]. Age distribution in HL has three incidence peaks, a small peak in childhood, a sharply rising incidence peak in adolescence and early adulthood (in developed countries), and a gradually increasing incidence after 50 years. cHL can be divided into four histologic subtypes, consisting of nodular sclerosing (NS), mixed cellularity (MC), lymphocyte rich (LR) and lymphocyte depleted (LD) [5].

Epstein-Barr Virus (EBV) has been shown to be clonally integrated in the malignant HRS cells in about 40% of cHL patients overall and ~20% of young adult nodular sclerosis cases (EBV+ cases) [1]. Prevalence of EBV involvement varies across age, sex, ethnicity and geographic location [6]. Latent EBV infection has B cell transforming potential and occurs early in the development of cHL, prior to clonal expansion of the malignant cells [7]. EBV infection in cHL involves a specific pattern of so-called latency type II viral protein production, limited to latent membrane protein 1 (LMP1), latent membrane protein 2 (LMP2) and EBV nuclear antigen 1 (EBNA) [8]. The presentation of peptides derived from these EBV proteins is mediated through human leukocyte antigens (HLA) classes I and/or II. Both cytotoxic T cells that recognize HLA class I [9,10] and T helper cells that react to HLA class II are involved in the anti-tumor immune response against EBV [11]. Genetic studies screening the entire HLA region and subsequent targeted studies have identified a clear role for the *HLA-A* gene in susceptibility to EBV+ cHL [12,13]. More specifically regarding HLA class I subgroups, the HLA-A*01 subtype of HLA I is positively associated with EBV+ cHL while the HLA-A*02 subtype is negatively associated with EBV+ cHL [14]. More recently, GWAS studies have shown that the *HLA-A* gene region is the dominant genetic susceptibility risk factor for EBV+ cHL, while loci in the class II region (*HLA-DRA*) are associated with EBV- cHL and NS cHL [15–17].

In multiple European studies, expression of HLA I and II on the cell surface of HRS cells is associated with EBV+ disease, suggesting that antigen presentation of EBV-derived peptides can occur in these tumor cells [18–20]. Both CD4 and CD8 T cells are in the close vicinity of the HRS cells and can presumably be activated by antigen presentation. In contrast, HLA class I and/or class II is downregulated in the HRS cells in many EBV- cHL cases in Western Europe [18–20].

In a study of Chinese cHL patients, HLA class I expression was positively associated with EBV status; whereas HLA class II was not [21]. HLA-typing of these Chinese patients showed that the HLA-A*02 type (which includes amongst others HLA-A*02:01 and HLA-A*02:07) was not associated with EBV+ cHL. This is explained by differential susceptibility effects of the specific HLA-A*02 suballeles. The European protective allele HLA-A*02:01 that accounts for the A*02 protective effect in Europeans is uncommon in Chinese and showed a trend for being protective. However, the more common Chinese-specific subtype HLA-A*02:07 was strongly associated with the risk of EBV+ cHL. Interestingly, the same allele proved to be protective for developing EBV- cHL in the Chinese [22]. Thus, differential genetic susceptibility across populations contribute to the well-known differences in incidence of EBV+ cHL in various parts of the world.

Hispanics (mostly of Mexican descent) comprise the largest non-white ethnic group in Los Angeles County [23]. California Hispanic cHL patients have a distinct disease pattern with a higher proportion of EBV+ and MC subtypes compared to non-Hispanic whites, and the HLA-A*01 frequency is intermediate (7% in Hispanics, 17% in non-Hispanic whites) [24]. Therefore, we examined HLA class I and II expression in relation to EBV status, the susceptibility effects of HLA-A*01 and A*02 (including A*02:01 and A*02:07) and other characteristics in a Hispanic population diagnosed at a large county hospital in Los Angeles County.

## Materials and methods

### Patients and tumors

Sixty-five Hispanic cHL patients of Mexican descent diagnosed at the Los Angeles County-University of Southern California (LAC-USC) Medical Center from 2000–2012 with sufficient tumor biopsy tissue were identified. Formalin-fixed paraffin-embedded tumor blocks were reviewed by a pathologist and areas marked for coring meeting the following criteria: sufficient amount of tumor, minimal sclerosis and presence of a minimum number of HRS cells (at least 3 present). Three 0.6mm cores were extracted from these areas in each block and used in tissue microarrays (TMAs).

### Patient characteristics

Pathology reports and patient charts from LAC-USC were reviewed and information on ethnicity, age at diagnosis, sex, histological subtype and birthplace were abstracted. The study was approved by the University of Southern California Institutional Review Board in accordance with the 1964 Declaration of Helsinki. There was no patient contact in this study; medical records and tumor blocks were linked and coded with numeric identification numbers. The individual patient data was stored on a separate password-protected server available only to key personnel and the personal identifiers necessary for linking the chart information to the tumors during the study period have now been removed, making the study anonymous.

### Immunohistochemistry

Five μm tissue slides were sectioned on APES coated slides, and deparaffinized utilizing xylene (2x10min) and ethanol (2x5min). The slides were rinsed with PBS before blocking with 0.3% H2O2 for 30 min. In-situ hybridization for EBV encoded RNA (EBER) was performed with the Novocastra^™^ Epstein-Barr virus ISH Kit using standard methods [14]. Monoclonal antibodies to the HLA-class I heavy chain (HC-10, kindly provided by prof. dr. J. Neefjes, the Netherlands Cancer Institute, Amsterdam, the Netherlands) and polyclonal rabbit anti human B2 microglobulin (B2M, DAKO, Glostrup, Denmark), at dilutions of 1:500 and 1:400 respectively, were used to mark HLA class I expression. Monoclonal antibody to CR3/43 (DAKO) that binds to the HLA-II chain of HLA-DP, HLA-DQ, and HLA-DR at a dilution of 1:200 was used to mark HLA class II expression. CD30 was used to aid in identifying HRS cells. Tissues were stained using the Leica Bond 3 staining platform. Scoring for EBER and HLA expression was performed if at least 50 HRS cells were present in all three cores combined. A case was considered positive for HLA if there was membranous staining in more than 50% of the HRS cells.

### HLA-A*01/A*02 quantitative PCR

Genomic (g) DNA was isolated from cHL FFPE tissue sections using ReliaPrep^™^ FFPE gDNA Miniprep System (Promega Corporation, Madison, USA) according to the manufacturer’s protocol. DNA concentration was measured with a Nanodrop^™^ 1000 Spectrophotometer (Thermo Fisher Scientific Inc., Waltham, USA). gDNA samples were diluted to a concentration of 4ng/μl for quantitative (q) PCR analysis.

qPCR analysis was performed in triplicate on an ABI PRISM 7900HT (Applied Biosystems^™^, Foster City, USA) with SYBR Green in a 384-well microtiter plate using primers for HLA-A*01 and HLA-A*02 (Table 1). Primers specific for the PTP4A1 gene were used as a quality and quantity control. Primer concentrations used for the reaction were as follows, 2μM for GP405, 1.5μM for GP406 and 3μM for the HLA-A*02 primers and the PTP4A1 primers. All reactions were performed in a final volume of 10μl consisting of 5μl SYBR^®^ Green PCR Master Mix (Applied Biosystems^™^), 1μl of each primer, 0.5μl of milliQ and 2.5μl of gDNA (4ng/μl). qPCR cycling conditions consisted of an initial 2 min. AmpErase UNG activation step at 50°C and a 10 min. hot start at 95°C followed by 40 cycles of denaturation at 95°C for 15s and combined annealing/extension at 60°C for 1 minute. A melt analysis was generated (95°C for 15s, 60°C for 15s and 95°C for 15s) to verify specificity of the PCR products. Each qPCR included no template controls (NTC) and 16 reference samples with known *HLA-A* genotype.

**Table 1:** Primers used for the HLA-A*01 and HLA-*02 quantitative PCR.

Specificity	Primer	Sequence* (5’–3’)	Location	Size
HLA-A*01	GP405	F:TCCGCGGGTACCGGCAGGAC	Exon 3	164bp
	GP406	R:GCACCGGCCCTCCAGGTAGA	Exon 3	
HLA-A*02	GP343	F:GAGCCCCGCTTCATCGCA	Exon 2	132bp
	GP344	R:CCCGTCCCAATACTCCGGA	Exon 2	
PTP4A1	PTP4A1F	F:GCACAGCACGACCTCTATGC	Exon 2	142bp
	PTP4A1R	R:CCAGGTCAGAACTCTTGTAAAATGC	Exon 2	

Note:

*F: forward primer, R: reverse primer

Mean cycle threshold (Ct) values for all genes were quantified by the SDS software (version 2.2). Samples with poor DNA quality were excluded based on a Ct value higher than 36 for PTP4A1. Relative expression levels were calculated as 2^-ΔCt^. Based on the 16 HLA-A typed samples, cut-off levels for HLA-A*01 positivity was set at a 2^-ΔCt^ value of 0.03 and for HLA-A*02 at a 2^-ΔCt^ of 0.1. To indicate the absence of HLA-A*02 or HLA-A*02 cut-off levels were set at a 2^-ΔCt^ value of 0.015 or 0.05 respectively.

Subtyping of HLA-A*02 positive individuals was performed by amplification and sequence analysis of exon 2 and exon 3 regions that contain a number of SNPs that discriminate between the common and well-documented (CWD) allelic variants in the Hispanic population [22].

### Statistical analysis

Associations between HRS cell HLA expression (Class I and Class II), HRS cell EBV status (positive [+] or negative [–]), histology (nodular sclerosis [NS] vs. other) and demographics (age, sex, birthplace) were evaluated using a Pearson Chi square test or Fisher’s exact test for expected cell counts < 5. A logistic regression analysis was conducted to assess the associations between HLA class I and class II expression and all characteristics using the Firth penalized likelihood approach to account for small sample size. Because HRS cell EBV status is associated with histological subtype, we adjusted for histology (NS vs. other) in the logistic regression analyses. Prevalence of the specific HLA Class I alleles HLA-A*01 and HLA-A*02 were compared between EBV+ and EBV- subgroups by Pearson Chi square and Fisher’s exact tests. For these analyses, the outcome was EBV tumor status and the exposure the specific germline HLA allele. All significance tests were two-sided and the significance level (α) was 0.05. In addition, we performed a power analysis to see whether we would have enough samples to find associations between HLA expression and HRS cell EBV status similar to the European population [20,25]. For HLA class I there was 99.8% power, for HLA class II this was only 29.8%. Analyses were performed with SPSS and SAS.

## Results

### Population characteristics

Population and tumor characteristics are shown in Table 2 and S1 Datatable. Of the 65 Hispanic patients, 47 (72.3%) were born outside the U.S. Thirty (46%) had EBV+ tumors. Histologically, 40 patients (61.5%) had NS, 19 (29.2%) had MC, 5(7.7%) had LD, and 1 (1.5%) had LR. There were more male (n = 47; 72.3%) than female (n = 18; 27.7%) patients.

**Table 2:** Characteristics of Hispanic classical Hodgkin lymphoma patients.

Characteristics	Number (%)
Total cases	65
Age	
• Mean	36.3
• Median	33.0
• Range	5–80
Sex	
• Female	18 (27.7)
• Male	47 (72.3)
Birthplace	
• US born	17 (26.1)
• Not US born	47 (72.3)
• Missing	1 (1.6)
EBV Tumor Status	
• EBV-	35 (53.8)
• EBV+	30 (46.2)
Histology	
• Nodular sclerosis	40 (61.5)
• Mixed cellularity	20 (30.8)
• Lymphocyte depleted	4 (6.2)
• Lymphocyte rich	1 (1.5)

### HLA expression and EBV association

Of the 65 cases, definitive HLA class I and HLA class II expression results were obtained for 50 and 44 patients’ tumors, respectively. HLA class I expression was positively associated with EBV+ cHL (p = 0.0006) (Table 3). There was no significant association between HLA class II expression and EBV+ cHL (p = 0.06) (Table 3). In a logistic analysis, EBV+ tumor cell status was significantly associated with HLA class I expression when adjusting for histology (Table 4, OR = 6.53, 95%CI = 1.80–23.72). EBV+ tumor cell status was not significantly associated with HLA class II expression (OR = 4.87, 95% CI = 0.75–31.69). HLA expression was not associated with any demographic characteristics. Relative to other histological subtypes, NS was not significantly associated with HLA class I (p = 0.08), or HLA class II (p = 0.7) expression (Table 3). Results were unchanged when the analysis was adjusted for EBV tumor status (data not shown).

**Table 3:** Association between HLA class I or II expression in Hodgkin Reed Sternberg cells and presence of Epstein-Barr Virus (EBV), Nodular Sclerosis (NS) histology, birthplace, sex and HLA type in Hispanic classical Hodgkin lymphoma patients.

	HLA class I		P	HLA class II		P
	Negative	Positive		Negative	Positive	
Sex						
• Female	8 (32%)	5 (20%)	0.33	1 (11%)	10 (28%)	0.41^a^
• Male	17 (68%)	20 (80%)		8 (89%)	25 (71%)	
Age (median)	27	37	0.65^b^	28	27	0.66^b^
Birthplace						
• Foreign born	17 (68%)	19 (79%)	0.38	6 (67%)	25 (71%)	0.78
• US born	8 (32%)	5 (21%)		3 (33%)	10 (29%)	
EBV						
• EBV-	20 (80%)	8 (32%)	0.0006	8 (89%)	18 (51%)	0.06^a^
• EBV+	5 (20%)	17 (68%)		1 (11%)	17 (49%)	
Histology						
• Non-NS	6 (24%)	12 (48%)	0.08	2 (22%)	13 (37%)	0.7^a^
• NS	19 (76%)	13 (52%)		7 (78%)	22 (63%)	
HLA-A*01						
• HLA-A*01-	20 (87%)	23 (92%)	0.66^a^	8 (89%)	29 (88%)	1^a^
• HLA-A*01+	3 (13%)	2 (8%)		1 (11%)	4 (12%)	
HLA-A*02						
• HLA-A*02-	18 (78%)	14 (58%)	0.14	6 (67%)	24 (75%)	0.68^a^
• HLA-A*02+	5 (22%)	10 (42%)		3 (33%)	8 (25%)	

^a^p-value based on Fisher's Exact test with two-sided alpha level of 0.05

^b^Mann Whitney U test.

All other p-values are based on Pearson Chi-squared tests.

**Table 4:** The association between EBV tumor status and HLA class I or HLA class II expression in Hispanic classical Hodgkin lymphoma patients.

EBV	HLA expression		OR_unadj_^a^	95% CI	OR_adj_^b^	95% CI^b^
	Negative	Positive				
HLA class I						
• EBV-	20	8	1.0	2.15–27.42	1.0	1.80–23.72
• EBV+	5	17	7.67		6.53	
HLA class II						
• EBV-	8	18	1.0	0.8–35.93	1.0	0.75–31.69
• EBV+	1	17	5.36		4.87	

^a^ Odds ratios estimated from logistic regression with Firth penalized likelihood approach

^b^ Adjusted for histology (Nodular Sclerosis vs. others).

### Specific *HLA-A* allele associations

We observed no association between HLA-A*01 or HLA-A*02 types and susceptibility to EBV+ cHL (Table 5, S1 Datatable). There was no association with expression of HLA class I (Table 3). All 19 HLA-A*02 positive patients carried the HLA-A*02:01 allele. Two patients carried a second HLA-A*02 suballele, one with A*02:02 and the other with A*02:06. The HLA-A*02:07 suballele was not present. Thus, in this study neither HLA-A*02 nor any specific HLA-A*02 suballele was associated with EBV+ cHL.

**Table 5:** The association between HLA-A*01 or HLA-A*02 and EBV tumor status in Hispanic classical Hodgkin lymphoma patients.

	EBV-	EBV+	P
HLA-A*01			
• HLA-A*01-	31 (91%)	22 (81%)	
• HLA-A*01+	3 (9%)	5 (19%)	0.44^a^
HLA-A*02			
• HLA-A*02-	24 (71%)	18 (67%)	
• HLA-A*02+	10 (29%)	9 (33%)	0.74^b^

^a^p-value based on Fisher's Exact test with two-sided alpha level of 0.05

^b^p-value based on Pearson Chi-squared test

## Discussion

This study is the first to examine associations between HLA and tumor cell EBV status in a Hispanic cHL population. Similar to previous studies in European and Chinese populations, HLA class I expression was strongly associated with EBV+ cHL. HLA class I expression by HRS tumor cells is quite consistent in European, Chinese and Hispanic cHL patients with percentages of 71%, 79% and 77% positivity in EBV+ and percentages of 14%, 30% and 29% positivity in EBV- tumors respectively [21,25]. It should be noted that scoring of membranous HLA class I expression in HRS cells is somewhat challenging as all nucleated cells normally express HLA class I. It helps to find areas in which tumor cells are adjacent, and in EBV+ cHL the expression in HRS cells is usually stronger than in surrounding reactive infiltrating cells. Nonetheless, we were able to reliably determine HLA class I expression in 50 out of the 65 patients.

Lack of membranous HLA class I has been attributed to mutations in beta-2-microglobulin (B2M) which prevents formation of the HLA class I heavy chain-B2M complex in HRS cells [26,27]. The high prevalence of HLA class I expression by EBV+ HRS cells in comparison to EBV- HRS cells is counterintuitive, as this implies that anti-EBV cytotoxic immune responses should be able to eradicate the tumor cells. On the other hand, retention of HLA class I expression may help the tumor cells in escaping from NK-cell mediated lysis [25]. In addition, HRS cells are well known for exhibiting various other immunomodulating mechanisms, like production of immunosuppressive cytokines, expression of PDL-1 and attraction of Th2 type T cells that can inhibit cytotoxic responses [1].

The protective effect of the HLA-A*02:01 allele in Europeans could be explained by common anti-EBV immune responses to antigens that are specifically presented by A*02:01 encoded HLA [28]. At the moment, it is unknown how HLA-A*01:01 and HLA-A*02:07 exert a risk effect, although this can be partially explained by lack of EBV latency type II responses in the context of these alleles. The risk effect of HLA-A*02:07 in Chinese EBV+ cHL patients coincides with an increased risk for another EBV latency type II driven cancer, undifferentiated nasopharyngeal carcinoma [29].

In the current study in Hispanics, we did not find a significant risk effect of HLA-A*01 or HLA-A*02:07 nor a significant protective effect of HLA-A*02 for EBV+ cHL. This might be explained by the relatively low frequency of A*01 and A*02 and the absence of A*02:07 in the Hispanic population. Indeed, given the measured allele frequencies in our Hispanic cohort, there is only about 50% power to detect the A*01 and the A*02 associations as found in the European population. Since we only interrogated the known risk and protective *HLA-A* alleles, it is possible that other HLA genes or suballeles are involved in susceptibility to EBV+ cHL in this population.

In comparison to HLA class I, HLA class II expression by tumor cells is somewhat more variable with 70%, 52%, 94% expression in EBV+ and 53%, 43%, 69% in EBV- tumors in Europeans, Chinese and Hispanics, respectively [20,21]. Although the HLA class II expression differences between EBV+ and EBV- cHL are not very pronounced, they do show a similar trend in all three populations and are significant in Europeans. Expression of HLA class II may be involved in establishment of the CD4+ T cell infiltrate in the cHL tumor microenvironment, however this is difficult to assess as it probably occurs early in disease pathogenesis [30]. Interestingly, HLA class II associations are mainly associated with EBV- cHL [15–17,31].

In conclusion, our data demonstrate that EBV+ cHL tumor cells more frequently retain HLA class I expression, regardless of ethnicity. Together with our previous studies, this study emphasizes that differences in ancestry should be considered in HLA susceptibility studies in cHL.

## Supporting information

S1 Datatable Characteristics and study findings of the cohort of Hispanic classical Hodgkin lymphoma patients. (XLS)
